# Changing QRS duration and morphology with A‐paced and A‐sensed rhythm in postcardiac resynchronization therapy (CRT) patients

**DOI:** 10.1111/anec.12703

**Published:** 2019-09-26

**Authors:** Saurabh Ajit Deshpande, Ameya Udyavar

**Affiliations:** ^1^ Department of Cardiology Jag Jivan Ram Western Railway Hospital Mumbai India; ^2^ Department of Cardiology P D Hinduja Hospital Mumbai India

**Keywords:** atrial pace, atrial sense, cardiac resynchronization therapy with defibrillation (CRT‐D), ischemic cardiomyopathy

## Abstract

Meticulous interpretation of the ECGs of patients with cardiac resynchronization defibrillator therapy (CRT‐D) device is important to ensure optimal synchronization of both the chambers. We present the ECGs of such a patient who was noted to have different morphologies of the biventricular‐paced QRS depending in atrial‐sensed (A‐sensed) or atrial‐paced (A‐paced) rhythm.

## 1 CASE REPORT

**Figure 1:**
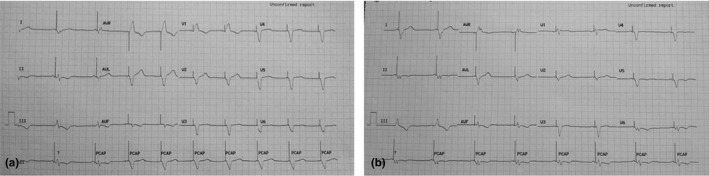
Post‐CRT ECGs (a) A‐paced BiV‐paced ECG with R in V1 and QRS duration of 200 ms. (b) A‐sensed BiV‐paced rhythm with RS pattern in V1 and QRS duration of 140 ms

A 61‐year‐old man, diabetic and hypertensive, presented with heart failure. He had a past history of myocardial infarction with left ventricular ejection fraction (LVEF) of 30%. He had left bundle branch block (LBBB) at baseline and was implanted with a cardiac resynchronization therapy with defibrillation (CRT‐D) device. During follow‐up after 6 months, his QRS morphology and duration were noted to change depending on whether it was A‐sensed or A‐paced rhythm (Figure 1). The programming parameters were kept at routine values (PAV of 110 ms, SAV of 90 ms, and VV of −30 ms [left ventricle—LV earlier]). Also, biventricular pacing was confirmed on device interrogation during both A‐sensed and A‐paced beats.

The first ECG (Figure 1a) shows an A‐paced rhythm with QRS duration (QRSd) of 200 ms, R wave in V1, deep S wave in V5 and V6, and RS pattern in lead II. While during A‐sensed rhythm (Figure 1b), the QRSd reduced to 140 ms, RS pattern in V1, smaller S wave in V5 and V6, and QS (W) pattern in lead II. This narrowing of the QRS and a better biventricular (BiV)‐paced morphology during A‐sensed rhythm will ensure better clinical outcome. This is essential to recognize as beta‐blocker used for therapy may cause more atrial pacing causing wider QRS.

## 2 DISCUSSION

Meticulous interpretation of the ECGs of patients with cardiac resynchronization defibrillator therapy (CRT‐D) device is important to ensure optimal synchronization of both the chambers. We present the ECGs of such a patient who was noted to have different morphologies of the biventricular‐paced QRS depending in atrial‐sensed (A‐sensed) or atrial‐paced (A‐paced) rhythm.

There may be multiple reasons for narrowing of the QRS and change in morphology with a A‐sensed beat. 1. The A‐paced rhythm will cause a delayed left atrial contraction causing decrease LV filling. 2. The morphology of the QRS suggests that A‐sense causes a better fusion of the QRS. This fusion might be due to an earlier intrinsic right bundle activation along with the LV‐paced activation. This was earlier noted by Bernheim et al. (2005).

Effective transvenous CRT requires, in patients with sinus rhythm, the proper detection of atrial signals and reliable atrial pacing, which is mandatory for AV‐synchronous ventricular pacing (Schuchert, Aydin, Israel, Gaby, & Paul, ). So, it is important to note the percentage of A‐paced beats so that beta‐blockers may need to be adjusted accordingly to ensure A‐sensed beats.

## 3 CONCLUSIONS

In a case of CRT, correct interpretation of the ECG is of paramount importance to ensure A‐sensed rhythm with a better fused QRS. So, it is prudent to allow the intrinsic atrial beats for the beneficial effects of CRT.

## 4 KEY LEARNING POINTS

- In follow‐up of patients who have undergone cardiac resynchronization therapy (CRT), meticulous ECG evaluation is important to evaluate the response to therapy.
- A‐sensed rhythm gives a better fused QRS as compared to A‐paced beat.
- So, it is beneficial to the patient to have A‐sensed BiV‐paced rhythm on follow‐up.
